# Epidemiology of acute kidney injury in Hungarian intensive care units: a multicenter, prospective, observational study

**DOI:** 10.1186/1471-2369-12-43

**Published:** 2011-09-13

**Authors:** Laszlo Medve, Csaba Antek, Balazs Paloczi, Szilvia Kocsi, Bela Gartner, Zsuzsanna Marjanek, Gabor Bencsik, Peter Kanizsai, Tibor Gondos

**Affiliations:** 1 Department of Anaesthesiology and Intensive Care Medicine, Dr. Kenessey Albert Hospital, Rakoczi ut 125-127, Balassagyarmat, 2660, Hungary; 2 Department of Anaesthesiology and Intensive Care Medicine, DEOC, Nagyerdei krt 98, Debrecen, 4032, Hungary; 3 Department of Anaesthesiology and Intensive Therapy, University of Szeged, Semmelweis ut 6, 6725, Szeged, Hungary; 4 Department of Anaesthesiology and Intensive Care Medicine, Petz Aladar County Hospital, Vasvari Pal ut 2-4, 9023, Gyor, Hungary; 5 Department of Anaesthesiology and Intensive Care Medicine, Javorszky Odon Hospital, Argenti Dome ter 1-3, Vac, 2600, Hungary; 6 Department of Anaesthesiology and Intensive Care Medicine, Hetenyi Geza County Hospital, Toszegi ut 21, Szolnok, 5004, Hungary; 7 Department of Anaesthesiology and Intensive Care Medicine, Szent Lukacs Hospital, Korhaz ut 39-41, Dombovar, 7200, Hungary; 8 Department of Oxyology and Emergency Care, Semmelweis University, Faculty of Health Sciences, Vas u 17, Budapest, 1088, Hungary

## Abstract

**Background:**

Despite the substantial progress in the quality of critical care, the incidence and mortality of acute kidney injury (AKI) continues to rise during hospital admissions. We conducted a national, multicenter, prospective, epidemiological survey to evaluate the importance of AKI in intensive care units (ICUs) in Hungary. The objectives of this study were to determine the incidence of AKI in ICU patients; to characterize the differences in aetiology, illness severity and clinical practice; and to determine the influencing factors of the development of AKI and the patients' outcomes.

**Methods:**

We analysed the demographic, morbidity, treatment modality and outcome data of patients (n = 459) admitted to ICUs between October 1^st^, 2009 and November 30^th^, 2009 using a prospectively filled in electronic survey form in 7 representative ICUs.

**Results:**

The major reason for ICU admission was surgical in 64.3% of patients and medical in the remaining 35.7%. One-hundred-twelve patients (24.4%) had AKI. By AKIN criteria 11.5% had Stage 1, 5.4% had Stage 2 and 7.4% had Stage 3. In 44.0% of patients, AKI was associated with septic shock. Vasopressor treatment, SAPS II score, serum creatinine on ICU admission and sepsis were the independent risk factors for development of any stage of AKI. Among the Stage 3 patients (34) 50% received renal replacement therapy. The overall utilization of intermittent renal replacement therapy was high (64.8%). The overall in-hospital mortality rate of AKI was 49% (55/112). The ICU mortality rate was 39.3% (44/112). The independent risk factors for ICU mortality were age, mechanical ventilation, SOFA score and AKI Stage 3.

**Conclusions:**

For the first time we have established the incidence of AKI using the AKIN criteria in Hungarian ICUs. Results of the present study confirm that AKI has a high incidence and is associated with high ICU and in-hospital mortality.

## Background

Despite improvements in the quality of critical care, the incidence and mortality of acute kidney injury (AKI) continues to rise during hospital admission [1-3]. AKI has been the focus of numerous publications and research projects in the past 5 years. The incidence of AKI (during hospital admission) ranges from 3 to 25% depending on criteria applied. The variety of definitions used in clinical studies may be partially responsible for the large variations in the reported incidence and the associated mortality (19-83%) of AKI [4-7]. Today the two widely accepted classification systems are the RIFLE criteria [8,9] and the staging system for AKI as established by Acute Dialysis Quality Initiative (ADQI) and the Acute Kidney Injury Network (AKIN), respectively [10]. Understanding the changing spectrum of AKI is necessary to facilitate quality improvement efforts and to design successful epidemiological trials.

We have no controlled data about the epidemiology of AKI in critically ill patients in Hungary. The aims of our study were to examine the incidence of AKI, to evaluate its impact, in the context of other risk factors, on outcomes (mortality, hospital- and ICU stay) and to compare the findings to the international experience.

## Methods

### Study participants

This study is a national, multicenter, prospective, epidemiological survey of AKI in 7 ICUs in Hungary. After ethical approval from each participating centre, we analysed demographic, morbidity and outcome data of 459 adult patients admitted to ICUs between October 1^st^, 2009 and November 30^th^, 2009. The participating centres represent the spectrum of Hungarian ICUs (each of them were multidisciplinary non-cardiac ICU, representing the occurrence ratio of ICU patients in Hungary): two university centres (University of Debrecen, University of Szeged), three regional hospitals (Petz Aladar County Hospital Gyor, Javorszky Odon Hospital Vac, Hetenyi Geza County Hospital Szolnok,) and two city hospital (Dr. Kenessey Albert Hospital Balassagyarmat, Szent Lukacs Hospital Dombovar). During the two months of the study, every newly admitted patient was registered in the survey and was followed up until hospital discharge or death in hospital.

Serum creatinine was determined at least once a day and urine output was recorded hourly, for all patients. AKI was defined and classified by the AKIN criteria [10], which has only three stages. Stage 1 is defined as an abrupt (within 48 hours) reduction in kidney function currently defined as an absolute increase in serum creatinine of more than or equal to 0.3 mg/dl (≥ 26.4 μmol/l), a percentage increase in serum creatinine of more than or equal to 50% (1.5-fold from baseline), or a reduction in urine output (documented oliguria of less than 0.5 ml/kg per hour for more than six hours). Stage 2 is defined as doubling of serum creatinine or a urinary output lower than 0.5 ml/kg/h for 12 h. Stage 3 is defined as tripling of serum creatinine or a serum creatinine higher than 4 mg/dl if there is an acute rise in serum creatinine of at least 0.5 mg/dl, or a urinary output lower than 0.3 ml/kg/h for 24 h, or anuria for 12 h. Stage 3 also includes patients who need renal replacement therapy, irrespectively of the stage they are in, at the time of renal replacement therapy.

Patients were categorized by serum creatinine and/or urine output into the AKIN stages and the highest AKIN stage during ICU staying was evaluated. Serum creatinine on ICU admission was used as a reference value, staging was based on the appropriate increase within the 48 hour observation. Declines in serum creatinine were not coded as AKI. Chronic kidney disease patients on dialysis (n = 3) and theoretically the renal transplant patients (n = 0) were excluded from the analysis. 12 patients had creatinine level > 300 μmol/L on admission (severe AKI or unrecognized chronic renal failure). These patients were involved into the analysis.

### Data collection

The Simplified Acute Physiology Score version II (SAPS II) [11] and the Sepsis-related Organ Failure Assessment Score (SOFA) [12] were used to evaluate severity of illness, and were calculated based on the worst variables recorded during the first 24 hours of ICU admission. The non-renal SOFA score was calculated from the total SOFA score minus the points for kidney insufficiency.

Multiple data were collected on each study participant, including: demographics, co-morbidities, hospital and ICU admission and discharge data, presumed aetiologies of AKI, surgical procedures, nonsurgical procedures, and renal replacement treatment modalities.

The aetiologies of AKI were identified from a group of seven possible choices (septic shock, hypovolemia, cardiogenic shock (these definitions were based on international guidelines), surgical procedure, obstructive nephropathies, drug-induced and others). Premorbid conditions were chosen from the following possibilities (medical or surgical admission; as well as cardiovascular, respiratory, gastrointestinal, neurological, and trauma diseases; malignancy, and others). More than one condition could be selected in each case.

The data were collected using an Excel-based data collection file. This was available to each participating centre with instructions. All centres were asked to complete the data entry and e-mail the data to the organisation centre. Upon arrival, all data were screened in detail by a dedicated intensive care specialist for any missing information, insufficient detail, or any other queries. Any queries generated an immediate e-mail inquiry with planned resolution within 48 h.

### Statistical analysis

All values were presented as mean values ± SD or as median with interquartile range (IQR) as appropriate. The mean values of the different groups were compared using two-sided t-test, the median values by the Kolmogorov-Smirnov test and the occurrence rates by the chi-square test. A forward stepwise logistic regression analysis (conditional) was perform to determine the independent risk factors for AKI and mortality The included variables were: age, gender, vasopressor requirement, AKI stages, SOFA, SAPS II, creatinine level at ICU admission and the maximum level during ICU staying, surgical/medical admission, the different diseases, sepsis, mechanical ventilation, as appropriate. All variables were deemed to be significant if *p *< 0.05. All analysis was performed by the SPSS statistical software package 15.0.

## Results

### Characteristics of the occurrence of AKI

Altogether 459 patients (aged 59.6 ± 16.2 years, male/female ratio 258/201) were entered into the study. Baseline characteristics of the patients are summarized in Table 1. Because there were no significant differences among the examined variables in respect of the gender, in the following analyses the data were drawn together. AKI patients (n = 112) tended to be older (64.9 vs. 57.6 years, p < 0.001) and usually had more severe underlying diseases (SAPS II. 47.5 vs. 22, p < 0.001, SOFA 6 vs. 2, p < 0.001). The proportion of patients who needed mechanical ventilation during their ICU stay differed significantly in patients without or with AKI (33.4% and 75.0%, p < 0.001) A similar difference was also observed in the vasopressor needs (12.1% vs. 51.8% in patients without and with AKI, respectively, p = 0.0018).

**Table 1:** Characteristics of patients during ICU staying

Parameters	All patient	AKI	non-AKI	p-AKI vs.non-AKI
Patient number n (%)	459	112 (24.4)	347 (75.6)	

Age (year), mean ± SD	59.6 ± 16.2	64.9 ± 14.4	57.6 ± 16.3	< 0001

SAPS II. score, median (IQR)	28 (16, 46)	47.5 (33, 59)	22 (14, 38)	< 0.001

SOFA score, median (IQR)	4 (1, 7)	6 (4, 9.25)	2 (1, 5.5)	< 0.001

SOFA_non renal_, median (IQR)		6 (3, 9)		

Se-creatinine at ICU admission (μmol/L), median (IQR)	76 (59, 103)	117.5 (81, 205)	70 (57, 87)	< 0.001

Se-creatinine peak-concentration (μmol/L), median (IQR)	80 (61, 112)	165.5 (112, 274)	71 (58, 89)	< 0.001

Mechanical ventilation, n (%)	200 (43.6)	84 (75)	116 (33.4)	< 0.001

Ventilator days, median (IQR)	3 (1, 7)	3.5 (2, 11)	2 (1, 7)	0.177

Vasopressor treatment, n (%)	100 (21.8)	58 (51.8)	42 (12.1)	0.002

Vasopressor hours, median (IQR)	49 (24, 96)	48 (24, 99)	63 (18, 92)	0.619

The incidence of AKI was 24.4%. By AKIN criteria 53 patients (11.5%) were in Stage 1, 25 patients (5.5%) in Stage 2 and 34 patients (7.4%) in Stage 3. Seventeen patients (15.2% of the AKI cases) had received renal replacement therapy.

The major reason for ICU admission was surgical in 64.3% (gastrointestinal tract surgery was the most common), followed by neurological, cardiovascular, pulmonary diseases and trauma cases (Table 2).

**Table 2:** Type and reason of ICU admissions

Reason for ICU admission	All patient *(n = 459)*	non-AKI *(n = 347)*	Acute kidney Injury				p-AKI vs. non-AKI
				
			Stage 1 *(n = 53)*	Stage 2 *(n = 25)*	Stage 3 *(n = 34)*	AKI all *(n = 112)*	
Surgical	295 (64%)	241 (69%)	27 (24%)	17 (15%)	10 (9%)	54 (48%)	< 0.001

Medical	164 (36%)	106 (31%)	26 (23%)	8 (7%)	24 (22%)	58 (52%)	

Cardiovascular	84 (18%)	60 (17%)	8 (7%)	6 (5%)	10 (9%)	24 (21%)	0.417

Respiratory	67 (15%)	57 (16%)	6 (5%)	0	4 (4%)	10 (9%)	0.086

Malignancy	126 (27%)	111 (32%)	9 (8%)	5 (4%)	1 (1%)	15 (13%)	0.002

Gastrointestinal	135 (29%)	92 (27%)	20 (18%)	12 (11%)	9 (8%)	41 (37%)	0.136

Neurological	85 (19%)	72 (21%)	11 (10%)	1 (1%)	1 (1%)	13 (12%)	0.067

Trauma surgery	50 (11%)	42 (12%)	6 (5%)	1 (1%)	1 (1%)	8 (7%)	0.184

Others	63 (14%)	42 (12%)	4 (4%)	7 (6%)	9 (8%)	20 (18%)	0.182

Case numbers and percentage ratios, according to the appropriate groups

**Figure 1:**
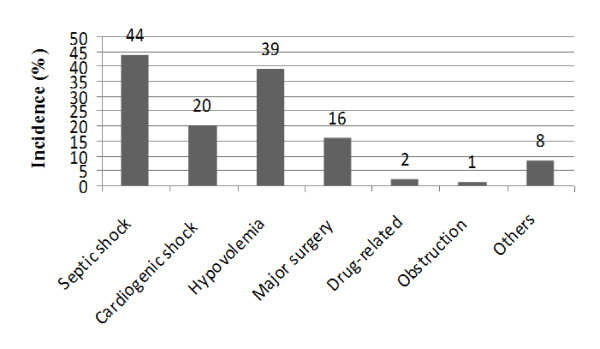
**Contributing Factors to Acute Kidney Injury**.

In 44.0% of patients, AKI was associated with septic shock (Figure 1). Sixteen percent of AKI was associated with major surgery, 20% was related to cardiogenic shock, 39% was related to hypovolemia, and 2% of AKI was potentially drug-related.

A logistic regression analysis was performed to analyse the predisposing factors for the incidence of AKI. Among the analysed parameters vasopressor treatment, SAPS II score, serum creatinine on ICU admission and sepsis were the independent risk factors for development of any stage of AKI (Table 3). The same distribution was found when the independent predisposing factors for AKI Stage 3 were evaluated.

**Table 3:** Stepwise logistic regression analysis of the variables for the incidence of AKI focused on AKI all and AKI Stage 3 versus non-AKI patients.

	Variables	Coefficient	SE	Wald test	*P *value	Odds Ratio	95% CI	
							
							lower	upper
AKI all	Vasopressor treatment	2.727	0.680	16.078	< 0.001	15.290	13.930	16.650
	
	SAPS II score	0.040	0.017	5.554	0.018	1.041	1.007	1.075
	
	Se creatinine at ICU admission	0.008	0.002	18.517	< 0.001	1.008	1.004	1.012
	
	Sepsis	1.980	0.544	13.225	< 0.001	7.242	6.234	8.250

AKI Stage 3	Vasopressor treatment	2.186	0.593	13.579	< 0.001	8.897	7.711	10.083
	
	SAPS II score	0.018	0.008	5.694	0.017	1.018	1.002	1.034
	
	Se creatinine at ICU admission	0.007	0.002	14.375	< 0.001	1.007	1.003	1.011
	
	Sepsis	1.574	0.500	9.907	0.002	4.827	3.827	5.827

SE, standard error; 95% CI, 95% confidence interval

### Renal replacement therapy

Among AKI Stage 3 patients 50% (17/34) received renal replacement therapy. The overall utilization of intermittent renal replacement therapy (IRRT) was high, with 64.8% (among all patients with RRT). All patients were treated with a veno-venous technique. The most common mode in the IRRT group was IHD (88%) and in the CRRT group CVVHDF (94%). The median filtration dose was a regimen of 20 mL/kg/h.

### Mortality and lengths of stay

The overall in-hospital mortality rate of AKI was 49.1% (55/112). The ICU mortality rate was 39.3% (44/112) (Table 4). Any degree of AKI was associated with a significantly increased all-cause ICU (39.3% vs. 9.5%, p < 0.001) and overall mortality (49.1% vs. 16.1% p < 0.001) compared with not having AKI. For patients admitted with AKI to the ICU, the median length of stay at the ICU increased by 120% (2 vs. 4.5 day, p < 0.0001) and the median length of hospitalization by 35% (10 vs. 13.5 day, p = 0.005), compared to patient without AKI. According to the logistic regression analysis (Table 5) age, mechanical ventilation, SOFA score and AKI Stage 3 were found as independent risk factors for ICU mortality. In respect of the hospital mortality only the higher age, the need of vasopressor treatment and the neurological diseases were the independent risk factors for the mortality.

**Table 4:** Mortality and length of stay

Parameters	All patients *(n = 459)*	non-AKI *(n = 347)*	Acute Kidney Injury				p-AKI all vs. non-AKI
				
			Stage 1 *(n = 53)*	Stage 2 *(n = 25)*	Stage 3 *(n = 34)*	AKI all *(n = 112)*	
ICU-s stay (days), median (IQR)	2 (2,5)	2 (2,4)	4 (2,8)	5 (3,14)	6 (2,18)	4.5 (2,13)	< 0.001

In-hospital stay (days), median (IQR)	11 (7,16)	10 (7,14)	14 (5,18)	12 (7,24)	14 (4,29)	13.5 (5,20)	0.015

Overall mortality, n (%)	111 (24.2)	56 (16.1)	19 (35.9)	11 (44)	25 (73.5)	55 (49.1)	< 0.001

ICU-s mortality, n (%)	77 (16.8)	33 (9.5)	14 (26.4)	10 (40)	20 (58.8)	44 (39.3)	< 0.001

In-hospital mortality after ICU, n (%)	34 (7.4)	23 (6.6)	5 (9.4)	1 (4.0)	5 (14.7)	11 (9.8)	0.301

**Table 5:** Stepwise logistic regression analysis of the variables for the ICU and hospital mortality based on the total population

	Variables	Coefficient	SE	Wald test	*P *value	Odds Ratio	95% CI	
							
							lower	upper
ICU mortality	Age	0.023	0.009	6.151	0.013	1.023	1.005	1.041
	
	Mechanical ventilation	1.976	0.371	28.382	< 0.001	7.217	6.475	7.959
	
	SOFA	0.212	0.042	25.572	< 0.001	1.236	1.152	1.320
	
	AKI Stage 3	1.317	0.439	9.012	0.003	3.731	2.853	4.609

Hospital mortality	Age	0.052	0.021	6.335	0.012	1.053	1.011	1.094
	
	Vasopressor treatment	1.481	0.532	7.763	0.005	4.398	3.334	5.462
	
	Neurological diseases	1.416	0.707	4.018	0.045	4.122	2.708	5.536

SE, standard error; 95% CI, 95% confidence interval

## Discussion

The increased incidence of AKI is most likely due to a trend of admitting older, more severely and more chronically ill patients to hospitals [7,13-16]. Different studies describe a wide range of AKI (5.2%-67.2%) [14,17-19], which varies across intensive care units and admission diagnoses [20-25]. There are two studies which assessed the prevalence according to the AKIN criteria [14,19] and have found it between 22%-35.5%, with different degrees of severity: AKI 1: 17.5%-19.1%, AKI 2: 2.4%-3.8% and AKI 3: 2%-12.5%. Joannidis M and co-workers analysed by AKIN criteria the SAPS 3 database, found the incidence 28.5%, that is very similar to our data (24.4%) [26]. While, they didn't found the difference between AKI 1 and 2, in our study AKI 1 (11.5%) was double to AKI 2 (5.5%). The difference may arise from the fact, that they assessing urine-output only at 24-hours intervals and could not distinguish between the AKIN stage 1 and 2. We found that the incidence of 7.4% in AKI 3 is better comparable to the data of Osterman [14] and Thakar [19], than the incidence of 13.8% of a cohort analysis [26].

We could demonstrate that the incidence rate of AKI is highest in elderly patients, who make up an ever-growing segment of the population. In the aging population, there is heightened susceptibility to drug toxicity, partially owing to altered drug pharmacokinetics and pharmacodynamics. Furthermore, elderly people consume twice as many medications overall, including nephrotoxic agents, than younger patients [16,27]. In our study, AKI patients were significantly older with significantly higher severity scores. We could prove that the higher SAPS II. score is an independent risk factor for AKI.

Renal dysfunction has a close association with the duration and weaning from mechanical ventilation [28]. Mechanical ventilation and vasopressor requirements were significantly higher in patients with AKI than in patients without AKI. Although, we could not distinguish that the mechanical ventilation or the vasopressor treatment was a cause or a consequent of AKI, we found a close relationship between them. Vasopressor treatment was a risk factor for the development of any stages of AKI, while mechanical ventilation was an independent risk factor for ICU mortality.

AKI has long been recognized as a devastating complication after surgery [3,29] and postoperative AKI remains a leading cause of morbidity, mortality and prolonged hospital stay [18,21,29-32]. Despite the widespread recognition of increased risk for AKI following a variety of surgical procedures, the pathogenesis of this syndrome is poorly understood in all of these settings. We can estimate that approximately thirty percent of AKI was associated with abdominal surgery [29,30]. Inadequate renal perfusion pressure caused by complicated abdominal surgery is one of the key factors in the development of intraabdominal pressure (IAP)-induced kidney injury. Changes in IAP may have a great impact on renal function and urine output. Currently available data on AKI and its consequences suggest that AKI has the potential to substantially alter the outcome of patients with cancer [33]. The high incidence of abdominal surgery (37%) and malignancy (13%) in our patients with AKI indicates a major role of these conditions in the development of AKI.

The multicentre European Sepsis Occurrence in Acutely Ill Patients (SOAP) study [34] found that 51% of septic patients developed AKI. More recently, in a 1-day point prevalence survey for severe sepsis/septic shock from 454 ICUs in Germany, Oppert and co-workers [35] reported concomitant AKI in 41.4% of septic patients. Likewise, two large multicentre observational studies of critically ill patients with AKI found sepsis to be a contributing factor in 46% to 48% of episodes of AKI [3,35]. In our study, sepsis was also the leading etiologic factor (in 44% of patients) of AKI, and was a highly significant independent risk factor for AKI.

Cardiogenic shock associated renal hypoperfusion is strongly associated with AKI. The incidence of acute cardio-renal syndrome in patients with AKI is estimated to be between 19% and 45% [36]. In a large cohort study of patient after cardiac arrest [37], it was found that AKI occurs in nearly 50% of patients when the AKIN criteria were applied. This indicates that the severity of hypoxia/ischemia may also have an effect on the development of AKI. Marenzi et al [38] found that in the setting of ST-elevation acute myocardial infarction, complicated by cardiogenic shock, AKI occurred in 55% of patients. In our study 20% of AKI was related to cardiogenic shock.

Hypovolemia-associated prerenal failure, an important contributing factor to AKI was noted in 36% of our AKI patients. Since our study protocol did not require an invasive hemodynamic monitoring, we could not assess exactly the volume-status of patients on ICU-admission. We evaluated it as a major limitation of our study because the diagnosis of hypovolemia was based mainly on basic hemodynamic data and clinical impressions. However, AKIN criteria do not determine specific diagnostic criteria to classify prerenal conditions. Because of the lack of standardized definitions and the difficulty in assessing reversibility of AKI, the concept of prerenal failure has been recently challenged [39].

It is known, that primary renal failure, secondary to a variety of pathologic conditions affecting the kidney, is usually treated in non-intensive care units and has relatively good prognosis and low mortality (5-10%) [40,41]. In contrast, the onset of AKI, as a part of multiple organ failure has 50-70% mortality [5,18]. Osterman et al [14] report that applying AKIN criteria, the mortality of AKI is the following: AKI 1: 20.1%, AKI 2: 25.9%, AKI 3: 49.6%. In our study the mortality was much higher: AKI all 41.3%, AKI 1 35.8%, AKI 2 44.0%, AKI 3 73.5%). The higher mortality of AKI in Hungarian ICUs can be explained also by factors, such as higher incidence of malignancy and non-uniform treatment principles. In the cohort analysis of Joannidis et al [26] 6.1% of AKI was related to non-metastatic cancer patients, while in our study it was 13%. The other reason may be that in Hungary there are not uniform protocols for treatment of AKI (different: the timing of RRT, the treatment modalities and doses of RRT, continuous vs. intermittent diuretic therapy). In our study the age, the sepsis related variables (vasopressor treatment, mechanical ventilation, SOFA score) and the AKI Stage 3 were the highly significant risk factors for the ICU and the hospital mortality.

The limitations of our study: Hypovolemia was not exclusion criteria in classification by AKIN. We applied early goal directed volume resuscitation, while followed the urine output hourly. If during the first six hours period the urine output raised above 0.5 ml/kg/h we didn't classified the patient for AKI 1 stage. Secondly, the Hungarian ICU-s hasn't uniform protocol for treatment of AKI, so we couldn't compare the different ICU-s. Finally, we didn't distinguish early and late AKI.

## Conclusion

We have conducted a national, multicenter, prospective, epidemiological study on AKI, occurring at representative Hungarian ICUs. For the first time, we have established the incidence of AKI using the AKIN criteria at Hungarian ICUs. The results of the present study confirm that AKI has a high incidence and is associated with higher ICU and in-hospital mortality. AKI doubles length of stay at the ICU and the duration of hospital staying. The independent risk factors for the development of any stage of AKI were SAPS II score, serum creatinine on ICU admission, sepsis and vasopressor treatment. Age, sepsis related variables (vasopressor treatment, mechanical ventilation, SOFA score) and AKI Stage 3 were the highly significant risk factors for the ICU and the hospital mortality.

## Abbreviations

ADQI: Acute Dialysis Quality Initiative; AKI: acute kidney injury; AKIN: acute kidney injury network; CRRT: continuous renal replacement therapy; CVVHDF: continuous veno-venous hemodiafiltration; ICU: intensive care unit; IHD: intermittent hemodialysis; IRRT: intermittent renal replacement therapy; RRT: renal replacement therapy; SAPS II: simplified acute physiology score; SOFA: sepsis-related organ failure assessment

## Competing interests

The authors declare that they have no competing interests.

## Authors' contributions

ML, ACS, PB, KSZ, GB, MZS, BG and KP collected the data, ML and GT drafted the manuscript. GT performed the statistical analysis. All authors read and approved this manuscript.

## Pre-publication history

The pre-publication history for this paper can be accessed here:

http://www.biomedcentral.com/1471-2369/12/43/prepub
